# Hyphal ontogeny in *Neurospora crassa*: a model organism for all seasons

**DOI:** 10.12688/f1000research.9679.1

**Published:** 2016-11-30

**Authors:** Meritxell Riquelme, Leonora Martínez-Núñez

**Affiliations:** 1 Department of Microbiology, Centro de Investigación Científica y de Educación Superior de Ensenada (CICESE), Ensenada, Baja California, 22860, Mexico

**Keywords:** microtubule, apical growth, filamentous fungi, polarity

## Abstract

Filamentous fungi have proven to be a better-suited model system than unicellular yeasts in analyses of cellular processes such as polarized growth, exocytosis, endocytosis, and cytoskeleton-based organelle traffic. For example, the filamentous fungus *Neurospora crassa* develops a variety of cellular forms. Studying the molecular basis of these forms has led to a better, yet incipient, understanding of polarized growth. Polarity factors as well as Rho GTPases, septins, and a localized delivery of vesicles are the central elements described so far that participate in the shift from isotropic to polarized growth. The growth of the cell wall by apical biosynthesis and remodeling of polysaccharide components is a key process in hyphal morphogenesis. The coordinated action of motor proteins and Rab GTPases mediates the vesicular journey along the hyphae toward the apex, where the exocyst mediates vesicle fusion with the plasma membrane. Cytoplasmic microtubules and actin microfilaments serve as tracks for the transport of vesicular carriers as well as organelles in the tubular cell, contributing to polarization. In addition to exocytosis, endocytosis is required to set and maintain the apical polarity of the cell. Here, we summarize some of the most recent breakthroughs in hyphal morphogenesis and apical growth in *N. crassa* and the emerging questions that we believe should be addressed.

## Introduction

Polarity is the key feature in all living cells and allows the asymmetrical transport of components to precise cellular sites. Filamentous fungi present one of the most extreme cases of polarized tip growth, among the different cell types that also extend apically (for example, pollen tubes, neurons, and root hairs). Fungal tubular cells, so-called hyphae, extend by continuous expansion of their apices through a complex mechanism that involves the coordinated directional supply of material needed for plasma membrane (PM) and cell wall growth. This process, hyphal morphogenesis, has increasingly been in the spotlight of fungal biology research in recent years.

*Neurospora crassa*, “the orange bread mold” discovered in French bakeries in the 19th century ^1^, has been at the vanguard of biochemistry, genetics, and biological research for nearly a century. Several reviews have highlighted the historical contribution of *N. crassa* to many areas of biology, including pioneering work on DNA silencing and circadian rhythms ^2, 3^. The genetic basis of the transition from single spherical conidia (asexual spore) to a large network of filamentous tubular hyphae ^4^ has been at the forefront of fungal biological research, elucidating both fungal morphogenesis and polarized growth. Most research aimed at identifying key players in hyphal morphogenesis before the availability of genome data involved forward genetics screens—bottom-up approaches—in which randomly generated mutants were analyzed to discover the function of a gene. This in fact was the basis for the leading work of the Nobel laureates George Beadle and Edward Tatum that established the relationship between genetics and biochemistry ^5^. When this strategy was followed, many *N. crassa* morphological mutants were generated ^6^.

The increasing availability of sequenced genomes made it possible to apply reverse genetics screens—top-down directed approaches—to silence or mutate specific genes and evaluate the resulting phenotypes. Sequencing of the *N. crassa* genome ^7, 8^, along with other key developments (expression plasmids for protein tagging ^9, 10^, recipient strains deficient in non-homologous end joining ^11^, and knockout cassettes for all annotated open reading frames ^12, 13^), revolutionized the field of fungal biology and quickly accelerated the number of studies on *N. crassa*, which set out to analyze the distribution, dynamics, and function of subcellular components ^14^. Needless to say, this led to significant advances in our understanding of fungal morphogenesis.

Research in genetics, biochemistry, and more recently “omics” conducted on *N. crassa* clearly contributed to many important insights into how fungal hyphae are shaped. This review focuses on the most recent findings on key subcellular structures determining hyphal ontogenesis in *N. crassa*.

## Hyphal morphogenesis

*N. crassa* exhibits a variety of cell morphologies corresponding to different developmental stages. The morphogenetic changes initiate when a conidium begins to grow isotropically during the first hours of hydration; soon after, the symmetry is broken, growth becomes polarized, and the resulting germ tube continues extending by apical polarized growth until it becomes a fully mature hypha. Further branching from subapical compartments generates new hyphal tips capable of fusing with each other and generating a mycelium ^6, 15– 18^.

Some remarkable differences in growth and intracellular organization have been described between germ tubes and mature vegetative hyphae in *N. crassa.* The most prominent characteristic of the apex in mature hyphae of *N. crassa* is the Spitzenkörper (Spk), a conspicuous accumulation of vesicles, ribosomes, actin microfilaments, and amorphous material of undefined nature ^19^. During the early stages of development, no Spk can be perceived at the germling apex ^20^ and this is most likely because of the insufficient number of tip-directed secretory vesicles ^21^. In addition, organelle distribution is disorganized in germlings and cytoplasmic microtubules (MTs) are less abundant, shorter, and differently distributed compared with mature hyphae ^20^. A widely accepted fungal morphogenesis model proposed that the Spk behaves as a vesicle supply center (VSC) ^22^. According to this model, the forward advancement of the VSC and the concomitant release of vesicles would generate an ideal hypha ^23^. An alteration of the number of released vesicles, the rate of advancement of the VSC, or a sustained displacement of its central position would generate several cell shapes, including branches and meandering hyphae ^24^. Growth of *N. crassa* in confined microfluidic structures, which mimic some of the characteristics in the natural environment, has allowed analysis of the thigmotropic response of individual hyphae and tip growth to changes in the environment ^25^ and has enabled long-term tracking to monitor, in real time, fluorescent reporters of molecular mechanisms such as circadian rhythms ^26^. In germlings with structures associated with cell fusion, so-called conidial anastomosis tubes (CATs), the displacement of activated GTPase clusters initiates repositioning of the apical secretory vesicle delivery machinery in response to chemotropic cues, offering an explanation of how directional tip growth is accomplished in cell types that lack a Spk ^27^.

**Figure 1.**
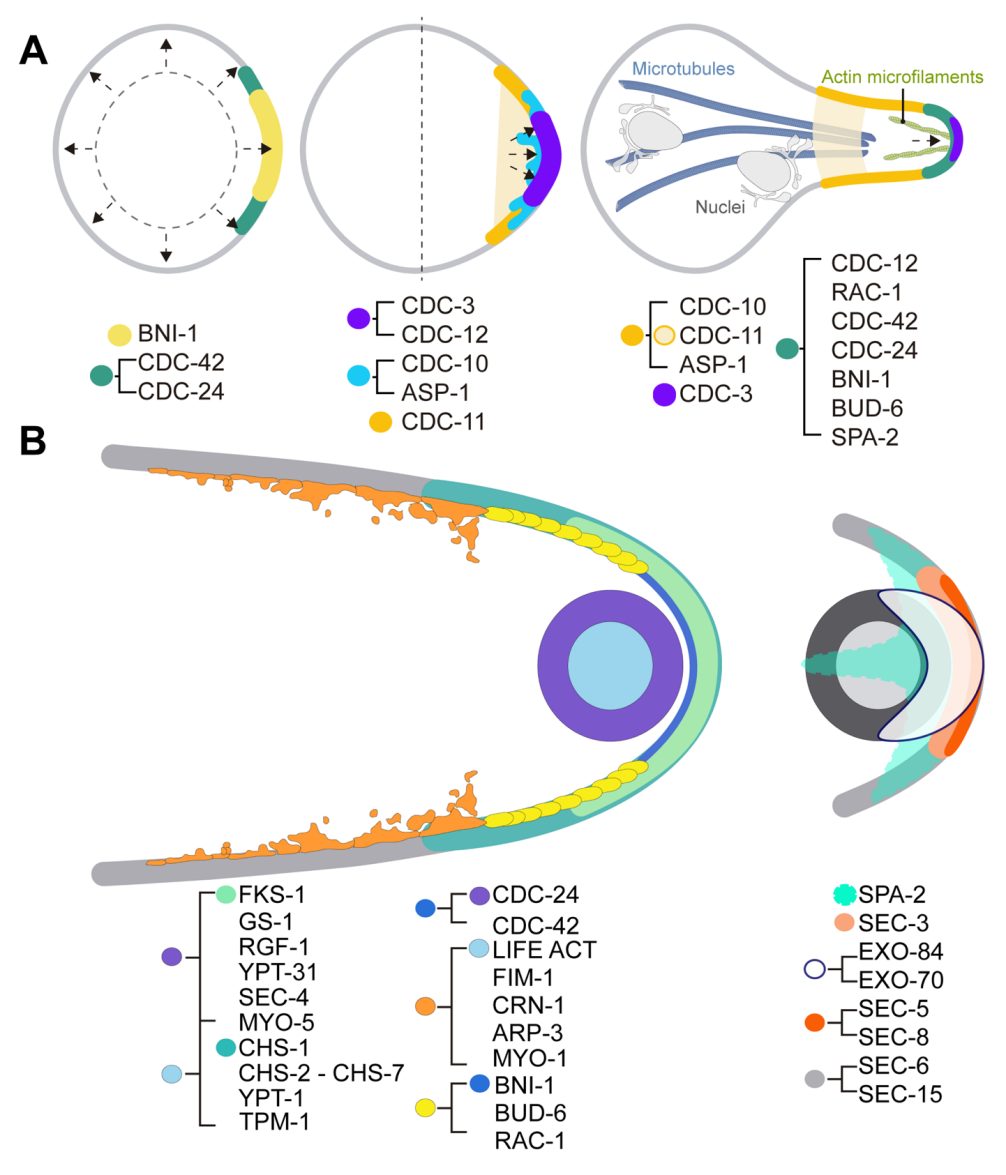
Representation of the localization pattern of proteins participating in hyphal morphogenesis in *Neurospora crassa*. ( **A**) Spatial distribution of the polarisome components (SPA-2, BUD-6, and BNI-1), Rho GTPases (RAC, CDC-42, and CDC-24), and septins (CDC-3, CDC-10, CDC-11, CDC-12, and ASP-1) during early developmental stages. ( **B**) Spatial distribution of the polarisome, Rho GTPases, GEFs (CDC-24 and RGF-1), Rab GTPases (YPT-1, YPT-31, and SEC-4), septins (CDC-3, CDC10, CDC11, CDC12, and ASP-1), actin-binding proteins (TPM-1, LIFE ACT, FIM-1, CRN-1, ARP3, MYO-1, and MYO-5), exocyst components (EXO-70, EXO-84, SEC-3, SEC-5, SEC-6, SEC-8, and SEC-15), and cell wall biosynthetic enzymes (FKS-1, GS-1, and CHS-1 to CHS-7) participating in apical extension of mature hyphae. Each protein displays one or two color tags corresponding to a specific distribution pattern.

Prior to symmetry breakage of an *N. crassa* spore, there is accumulation and localized activation of the small Rho GTPase CDC-42 and its guanidine exchange factor (GEF) CDC-24 ^27, 28^. Once a polarized germ tube has emerged, a second RHO GTPase, RAC, is recruited at the incipient tip forming a crescent ^28^. CDC-42 and RAC regulate the negative chemotropism exhibited during germ tube development and the positive chemotropism observed during CAT formation and cell fusion ^27^. In mature hyphae, CDC-42, CDC-24, and RAC are localized at the apical dome ^28^ ( Figure 1A). The homologues of the polarisome components BUD-6 and SPA-2 are required for the maintenance of apical growth and cell morphology of *N. crassa* germlings and hyphae but not for cell symmetry breakage during conidial germination ^28, 29^. SPA-2 accumulates at apices of hyphae adopting a fan shape, whose base co-localizes partially with the Spk core ^30^. BUD-6 accumulates at the apical PM, excluding the very tip. In addition, the formin BNI-1 (an F-actin polymerization protein), another polarisome putative component, has a distribution similar to that of BUD-6 but also co-localizes with the Spk ^29^ ( Figure 1A). Polarisome proteins are possibly involved in the thigmotropic response of *N. crassa* by mediating the reorientation of actin microfilaments, thereby regulating the vectorial flow of vesicles to the tip ^31^. It has been proposed that to convey information from the sensing apex to the transcriptional machinery that is located several micrometers behind the tip (11 μm in *Aspergillus nidulans*, 12 μm in *N. crassa*, and 22 μm in *Ustilago maydis* ^32– 34^), a signal is able to travel in the opposite direction from growing tips to the nuclei ^33^.

## Building the cell wall

Fungal hyphae are surrounded by a wall made of polysaccharides and glycoproteins, which helps the cell withstand internal pressure and serves as an external barrier against environmental stresses. It is therefore essential for the optimal development and survival of fungi. In *N. crassa*, the cell wall comprises an inner layer of chitin (9%) and β-1,3-glucans (87%) microfibrils, embedded in a matrix-like material composed of galactomannans, mixed traces of β-1,4-glucans, glucuronic acid, and glycoproteins ^35– 39^. These elements are enzymatically cross-linked to form a three-dimensional network.

The cell wall is synthesized at the apex and its assembly is responsible for hyphal shape ^40^. Chitin is synthesized by a family of chitin synthases (CHSs), CHS-1 to CHS-7, in *N. crassa* ^41^. These transmembrane proteins take N-acetylglucosamine subunits (Glc-NAc) from a UDP-Glc-NAc donor and polymerize them into a linear chain of chitin (β-1,4-Glc-NAc) ^36^. β-1,3-glucans are manufactured by a glucan synthase complex (GSC) composed of FKS-1 (catalytic subunit), RHO-1 (regulatory subunit), and a third protein, GS-1 (orthologous to Knr4p/Smi1p in *Saccharomyces cerevisiae*), required for β-1,3-glucan synthase activity ^42, 43^. The GSC takes glucose (Glc) subunits from the cytoplasmic donor UDP-Glc and extends them into a growing chain of β-1,3-glucans. Chitin and β-1,3-glucans are extruded as linear polysaccharides into the cell wall space, where they are modified by cross-linking enzymes ^36, 44^.

α-1,3-glucans have been described as an important component of the fungal cell wall ^45, 46^, but in *N. crassa* these polysaccharides have been detected in macroconidia only ^47^. Additional proteomic analysis indicated compositional differences between cell walls of conidia and hyphae, suggesting differential regulation of polysaccharide biosynthesis through development ^48^. Mannan and galactomannan are found as glycoconjugates on cell walls. The α-1,6-mannosyltransferase OCH-1 and the α-1,6-mannanases DFG-5 and DCW-1 are required for the efficient covalent incorporation of glycosylated proteins into the cell wall ^37, 44^. Many of these proteins are found attached to the PM by a glycosylphosphatidylinositol (GPI) anchor, an indispensable post-translational modification for optimal cell wall function in *N. crassa* ^49^.

**Figure 2.**
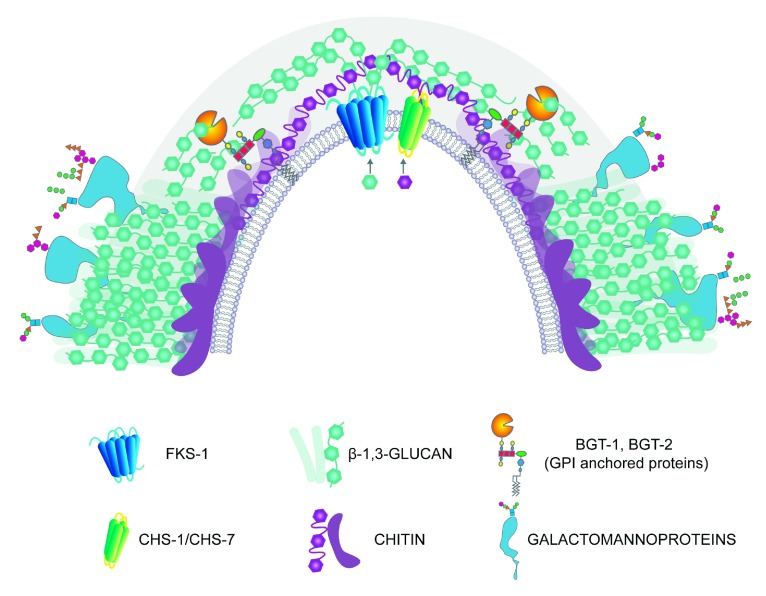
Apical distribution of the cell wall biosynthetic nanomachinery. FKS-1 and CHS are transmembrane proteins that take precursors from the cytoplasm to incorporate them into a growing chain of β-1,3-glucan or chitin, respectively. The polysaccharide chains are extruded to the cell wall and remodeled by glycosylphosphatidylinositol (GPI) glycosyltransferases that hydrolyze the chains and transfer the cleaved residues to another chain of β-1,3-glucan or chitin. Polysaccharide chains are cross-linked to cell wall glycoproteins.

A few decades ago, the unitary model of cell wall growth proposed that cell wall construction during apical extension requires a delicate balance of exocytic vesicles carrying lytic enzymes for cell wall softening as well as simultaneous secretion of vesicles transporting synthesizing enzymes ^50^. Subsequently, the steady-state model introduced the concept of plastic cell wall material extruded to the apex as individual chains, which gradually become cross-linked at the subapex by glycosyltransferases, resulting in the hardening of the cell wall ^51^. The role of remodeling enzymes such as chitinases, glucanases, or glycosyltransferases has recently been addressed in *N. crassa*. To elucidate the role of glycosyl hydrolase (GH) family 18 in *N. crassa* growth, deletion strains for the corresponding genes were phenotypically characterized. The lack of GPI-anchored chitinase CHIT-1 led to a decrease in growth rate, suggesting a potential role of chitinases in hyphal extension ^52^. Subcellular distribution analysis of chitinases has not been carried out in *N. crassa*. However, recently, two GPI-anchored β-1,3-endoglucanases, BGT-1 and BGT-2, with predicted glycosyltransferase activity (GH-17) were reported at cell wall remodeling sites (that is, hyphal apices and septa) during *N. crassa* vegetative development ^53^. These observations and previous attempts to reconcile the above-mentioned models ^54^ led to a new, integrated model of cell wall assembly. Remodeling enzymes, such as β-1,3-endoglucanases with glycosyltransferase activity that are anchored to the PM, hydrolyze long chains of β-1,3-glucans (at the tip) and transfer the cleaved residues to other chains. The new amenable free ends generated can be further cross-linked with chitin or glycoproteins, thus contributing to the construction of new cell wall ^53^ ( Figure 2). Nonetheless, further analysis of the cellular distribution of different cell wall remodeling enzymes is needed to attain a more comprehensive understanding of the process.

## The apical machinery

**Figure 3.**
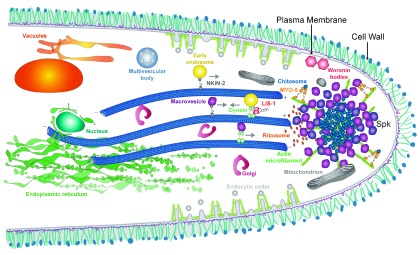
Depiction of a *Neurospora crassa* hyphal apex and subapex with some of the components participating in polarized growth. Vesicles move along microtubules or actin microfilaments, helped by motor proteins, to reach the Spitzenkörper (Spk). There, vesicles accumulate prior to fusing to the plasma membrane via exocytosis. At the subapex, an actin collar mediates endocytosis and recycling of important polarity factors. Some representative organelles are shown.

Decades after the discovery of the Spk in *Coprinus* fixed cells using iron-hematoxylin staining and in living hyphae of *Polystictus versicolor* by phase-contrast microscopy ^55, 56^, the pivotal role of the Spk in maintaining apical growth and determining hyphal growth direction and morphology was confirmed ^19^. Hence, it was predicted that the vesicles that comprise the Spk carry cell wall biosynthetic enzymes ^50, 57^. This hypothesis has been proven correct in the past few years. In *N. crassa*, all CHSs (CHS-1 to CHS-7) accumulate at the core of the Spk ^58– 60^, whereas FKS-1, GS-1, and RGF-1 (RHO-1 GEF) accumulate at the periphery of the Spk ^61– 63^. The localization pattern of CHS and the GSC in the inner and outer regions of the Spk corresponds to the microvesicular (chitosomes) and macrovesicular regions of the Spk ( Figure 1B and Figure 3), revealed by transmission electron microscopy in *N. crassa* and by electron tomography in *A. nidulans* ^64, 65^. Small RAB GTPases mediate the traffic of vesicles. In *N. crassa*, YPT-1 ^Rab1^ was detected in the Spk microvesicular core, whereas SEC-4 ^Rab8^ and YPT-31 ^Rab11^ occupied the Spk macrovesicular peripheral layer, implying different regulatory pathways for each type of vesicle ^66^ ( Figure 1B). In *A. nidulans*, RabC ^Rab6^ and RabO ^Rab1^ were also found at the Spk, although their localization could not be correlated to a specific population of vesicles ^67, 68^. Recent evidence demonstrated that the *A. nidulans* GS-1 homologue GsaA also accumulates at the outer region of the Spk ^69^. Remarkably, the flippases DnfA and DnfB, which regulate phospholipid asymmetry and presumably membrane bending, occupy the outer and inner regions of the Spk in *A. nidulans*, respectively, a finding that supports differences in the nature of the Spk secretory vesicles ^69^. In contrast, Chs6, a class VII CHS in the maize pathogen basidiomycete *U. maydis*, is transported in vesicles carrying Mcs1, a class V CHS with an N-terminal myosin motor domain similar to that of myosin-5 ^70^. Also, Gsc1, the *U. maydis* homologue of Fks1, is delivered in Mcs1-containing vesicles. *U. maydis*, however, does not display an Spk in any of its yeast or hyphal forms and has growth rates several orders of magnitude lower than those of *N. crassa*.

The short-distance delivery of vesicles from the Spk to the cell surface requires the orchestrated action of the exocyst complex, which tethers vesicles to the PM before their soluble *N*-ethylmaleimide sensitive factor attachment protein receptor-dependent fusion at specific PM sites. In *N. crassa*, the exocyst consists of eight proteins that seem to be essential for viability, except for SEC-5, whose loss resulted in impaired secretion and aberrant morphology ^71^. SEC-3 accumulates between the Spk outer layer and the PM; SEC-5, SEC-6, SEC-8, SEC-10, and SEC-15 accumulate as a surface crescent in the apical PM; and EXO-70 and EXO-84 accumulate primarily in the Spk outer layer ^71^ ( Figure 1B). Thus, in *N. crassa*, all exocyst components were excluded from the microvesicular region, leaving an open question about the secretion mechanism followed by chitosomes from the Spk to the cell surface. In the rice pathogen *Magnaporthe oryzae*, the exocyst proteins Sec5 and Exo70 participate in an alternate exocytic pathway for cytoplasmic effector proteins destined to be delivered to the host cells during biotrophic invasion of rice ^72^.

## Of filaments and nanomotors: connecting exocytosis and endocytosis

A complex network of proteins organized in a variety of forms such as tubules, patches, filaments, rings, and scaffolds composes the cytoskeleton in fungal cells. Collectively, cytoskeletal elements participate in shaping the hyphae, in the positioning and shuttling of vesicles and organelles, and in cellular division. The large cells of *N. crassa* constitute an excellent experimental system to study in great detail the distribution, dynamics, and function of the main cytoskeletal components: MTs, actin, associated proteins, molecular motors, and septins.

Immunolocalization studies of cryo-fixed *N. crassa* cells revealed long prominent cytoplasmic MTs distributed longitudinally along the axis of growth of hyphae ^64^. Subsequently, live imaging by confocal laser scanning microscopy of *N. crassa* cells expressing green fluorescent protein (GFP)-tagged β-tubulin revealed with unprecedented detail the dynamics and three-dimensional organization of MTs ^73^. Often an intertwined network of helical cytoplasmic MTs and individual MTs grouped at times into bundles was observed ^73^. Although there is no direct evidence of vesicles moving along MTs, cumulative observations suggest tip-directed vesicle traffic via an MT-based mechanism until vesicles reach the Spk, where a switch to the actin cytoskeleton occurs ^65^. MTs extend to the hyphal apex, either terminating at the vicinity of the Spk or traversing it and reaching the apical PM ( Figure 3). When the Spk is displaced from its most forward position and growth temporarily ceases, a rearrangement of the long MTs into short segments and bundles of MTs surrounding the retracted Spk is observed and this supports the association suggested between the Spk (or its constituents) and MTs ^73^.

In *N. crassa*, both plus-ends and minus-ends are present at hyphal tips ^74^, indicative of MT mixed polarity. The dynamic plus-ends of MTs undergo rapid polymerization and depolymerization, a phenomenon known as dynamic instability ^74^. Analysis of MT dynamic instability in mature leading hyphae of *N. crassa* by total internal reflection fluorescence microscopy revealed very fast MT polymerization rates that depend on the role of molecular motors ^74^. Lis1, an MT plus-end-binding protein, is involved in the regulation of the dynein/dynactin complex in humans ^75^. In *N. crassa*, two paralogues of Lis1—LIS1-1 and LIS1-2—were identified in the hyphal apical dome as short filaments or comet-like structures decorating the terminal plus-ends of the MTs ^76^, similar to the distribution of the MT plus-end-binding protein MTB-3, a homologue of EB1 ^77^. LIS1-1 localization was found to be dependent on the minus-end-directed MT-associated motor dynein and dynactin (p150Glued) ^76, 78^. Importantly, the dynein-dynactin complex has a role not only in minus-end-directed cargo traffic but also in the organization of the MTs in *N. crassa* ^76^.

Within the superfamily of kinesins, 10 different kinesins have been reported for filamentous fungi ^79^. In *N. crassa*, conventional kinesin-1 KIN-1 (NKIN) is believed to be responsible for vesicular transport and also nuclear, mitochondrial, and vacuolar migration ^80^. However, an *N. crassa* strain expressing KIN-1-GFP displayed dispersed cytoplasmic fluorescence ^81^. Microscopy approaches with a better temporal and spatial resolution may be needed to resolve the traffic of these motors. Alternatively, another unexplored kinesin could be responsible for vesicular traffic. Kinesin-3 NKIN2 co-localized with YPT-52 ^Rab5^, indicating its involvement in the transport of early endosomes, as has been previously observed, for kinesin-3 motors in *U. maydis* and *A. nidulans* ^81– 83^. The role of NKIN2 in early endosome transport was confirmed in Nkin2 deletion mutants, where endosomal movement was strongly reduced ^81^. The shuttling of early endosomes in the opposite direction also depends on dynein ^81, 84– 86^. The role of early endosomes in apical growth is just starting to be disclosed. Early endosomes are much more than mere organelles originated from endocytosis. They are interconnected with other organelles of the secretory pathway and behave as another hub for sorting cargos (Golgi compartments have been traditionally considered the main sorting hub of the cell). For example, *U. maydis* and *A. nidulans* endosomes transport peroxisomes, endoplasmic reticulum, lipid droplets, and mRNAs ^85, 87, 88^. *N. crassa* has a peroxisome-derived organelle, the Woronin body (WB), which reseals membrane lesions by occluding septa ^89^. WBs are associated with the cell cortex and the cell apex via a Leashin tether that promotes WB inheritance and holds it in position until signals from cellular damage induce release, translocation to the septal pore, and membrane resealing ^90^ ( Figure 3).

Motor protein rigor mutants, with a point mutation in the ATP-binding site, bind to the cytoskeletal element but cannot hydrolyze ATP, and this ATP-bound form is irreversibly locked to its cytoskeleton partner ^91^. In *N. crassa*, NKIN2 ^rigor^-GFP was found to associate preferentially with a subpopulation of straight detyrosinated MTs, as reported earlier for the corresponding *A. nidulans* orthologue UncA ^83^. Although the role of post-translational modifications in MTs is not totally understood, it is possible that they serve as a traffic signal for specific organelles. Thus, the existence of different populations of MTs could ensure that the tip-directed transport of cargo along a subpopulation of MTs remains stable during mitosis, when most cytoplasmic MTs are disassembled ^92^.

Though not absolutely indispensable for polarized growth, MTs and MT-associated molecular motors are important for Spk stability and hyphal morphogenesis ^93^. Mutations or inhibitors leading to MT disorganization in *N. crassa* cause growth defects, distorted hyphal morphology, and erratic Spk movement while maintaining polarized growth ^64, 78^. In contrast, actin is essential for hyphal polarity. Direct tagging of G-actin with fluorescent proteins has not been possible in *N. crassa*. To circumvent this, several F-actin-binding proteins, including fimbrin (FIM), tropomyosin (TPM-1), coronin (CRN-1), ARP-3, and Lifeact, a 17-amino-acid peptide derived from *S. cerevisiae* Abd120p, were fluorescently tagged and imaged by live microscopy ^94– 97^. The Lifeact reporter uncovered different subpopulations of actin macromolecular structures, including rings, actin patches, and actin microfilaments. FIM, ARP-3, and CRN-1 were found to be associated with patches arranged in a cortical ring at the hyphal subapical region (1 to 4 μm behind the growing tip). Although now it is widely accepted that this subapical collar of actin patches delimits the region of endocytosis, at the beginning of the 21st century it was doubted that endocytosis occurred in filamentous fungi ^98^. Endocytosis and exocytosis are spatially coupled. Endocytosis at the subapical collar is thought to contribute to the recycling of apical receptors and cortical markers ^98^ since it has been proven necessary to establish polarity and to maintain apical growth ^96, 98– 101^. TPM-1 was identified at the core of the Spk and at actin cables departing from the Spk toward the subapex. Lifeact elucidated all forms of actin. Whereas F-actin is thought to mediate short-range movements, the long cables observed might serve as tracks along which myosin motors transport secretory vesicles to and within the apex ^94^ ( Figure 3). Although we focus on the hyphal apex in this review, it is noteworthy to point out that prior to the formation of the contractile actomyosin ring (CAR) at septation sites, a septal actomyosin tangle (SAT) was observed ^102^.

Myosins are another superfamily of molecular motors that move along actin microfilaments. In *N. crassa*, MYO-1 is a component of the actin subapical collar and is thought to have a role in endocytosis ^103^. MYO-2 is involved mainly in the formation of the SAT and CAR during cytokinesis ^102, 104^. MYO-5, found occupying all the Spk ^61^ ( Figure 1B), is thought to be involved in exocytosis and hyphal morphogenesis. The MYO-5 homologue in *A. nidulans*, MyoE, is needed for the accumulation of RabE ^Rab11^ post-Golgi carriers at the tip ^105^.

Finally, septins are cytoskeletal components with GTP and phosphoinositide-binding domains ^106^. *N. crassa* septins CDC-3, CDC-10, CDC-11, CDC-12, and ASP-1 accumulate at or close to the PM in germinating conidia at sites of symmetry breakage ( Figure 1A) ^107^. They are suggested to constrain and corral the polarity machinery to the apical PM, acting as a molecular boundary between the apex and the region behind ^107^.

## Future directions

There are several unresolved questions that need to be addressed to further develop a holistic view of *N. crassa* hyphal morphogenesis. We need to do the following: (a) identify the real triggers of polarity; (b) generate mathematical models to test whether the spatial segregation of the vesicles at the Spk provides any morphogenetic advantage; (c) elucidate the interaction, if any, between the MT and actin cytoskeletons; (d) investigate the role of the predicted hypothetical proteins encoded in the *N. crassa* genome in polarity establishment and hyphal morphogenesis; (e) discern whether endocytosis occurs at sites other than the subapical collar, where actin patches have also been found; (f) determine which enzymes are secreted by a non-conventional pathway depending on mRNA transport; and (g) investigate, within the hyphal apical compartment, which nuclei are responding to external signals sensed at the growing tip.

Something to take into consideration is that some of the findings obtained from research in *N. crassa*, while conserved among several fungal taxa, should not be extrapolated to all members of the fungal kingdom. There are innate differences at the subcellular level among fungal species that may reflect their evolutionary distance.

## Abbreviations

CAR, contractile actomyosin ring; CAT, conidial anastomosis tube; CHS, chitin synthase; CRN-1, coronin; FIM, fimbrin; GEF, guanidine exchange factor; GFP, green fluorescent protein; GH, glycosyl hydrolase; Glc, glucose; GPI, glycosylphosphatidylinositol; GSC, glucan synthase complex; MT, microtubule; NKIN, *Neurospora crassa* conventional kinesin-1; PM, plasma membrane; SAT, septal actomyosin tangle; Spk, Spitzenkörper; TPM-1, tropomyosin; VSC, vesicle supply center; WB, Woronin body.
